# Preparation of Perfluorosulfonated Ionomer Nanofibers by Solution Blow Spinning

**DOI:** 10.3390/membranes11060389

**Published:** 2021-05-25

**Authors:** Masahiro Shinkawa, Kazunori Motai, Keita Eguchi, Wataru Takarada, Minoru Ashizawa, Hiroyasu Masunaga, Noboru Ohta, Yuhei Hayamizu, Hidetoshi Matsumoto

**Affiliations:** 1 Department of Materials Science and Engineering, Tokyo Institute of Technology, 2-12-1 Ookayama, Meguro-ku, Tokyo 152-8552, Japan; smasa165@gmail.com (M.S.); motai.k.aa@m.titech.ac.jp (K.M.); eguchi.k.ae@m.titech.ac.jp (K.E.); takarada.w.aa@m.titech.ac.jp (W.T.); ashizawa.m.aa@m.titech.ac.jp (M.A.); hayamizu.y.aa@m.titech.ac.jp (Y.H.); 2 Japan Synchrotron Radiation Research Institute, 1-1-1 Kouto, Sayo, Hyogo 679-5198, Japan; masunaga@spring8.or.jp (H.M.); noboru_o@spring8.or.jp (N.O.)

**Keywords:** perfluorosulfonated ionomer, Nafion, nanofiber, solution blow spinning

## Abstract

In this work, we report the preparation of high-purity perfluorosulfonated ionomer (Nafion) nanofibers (NFs) via solution blow spinning (SBS). Fiber formation in solution jet spinning is strongly dependent on the structure of the spinning solution. Upon adding a small amount of poly(ethyleneoxide) (PEO) as a spinning aid to Nafion dispersion, most of the highly ordered Nafion aggregate disappeared, allowing the stable production of bead-free and smooth high-purity NFs (Nafion/PEO = 99/1) by SBS. The microstructure of the blowspun Nafion NFs differed from that of electrospun NFs. In the blowspun NFs, incomplete microphase separation between hydrophilic (ionic) and hydrophobic domains was observed, but the crystallization of CF_2_−CF_2_ chains was enhanced owing to the high extensional strain rate and rapid solidification during SBS. These findings provide fundamental information for the preparation and characterization of blowspun Nafion NFs.

## 1. Introduction

Perfluorosulfonated ionomers (e.g., Nafion^®^, Flemion^®^, Aciplex^®^-F) have attracted much attention as polyelectrolyte (proton-exchange) membranes in various fields, such as polymer electrolyte fuel cells (PEFCs), chlor-alkali cells, and water electrolyzers, owing to their excellent chemical stability and high proton conductivity [1,2,3,4]. Nafion, a commonly used commercial ionomer, is a random copolymer consisting of an electrically neutral semi-crystalline poly(tetrafluoroethylene) backbone and pendant side chains terminated by sulfonic acid groups (polysulfonyl fluoride vinyl ester) [5,6]. The backbone and pendant ionic groups are completely different in nature; thus, they spontaneously form a microphase-separated structure. This structure allows the ionic domain to swell in the presence of water or other solvent molecules, particularly under high humidity conditions. The swelling results in the emergence of efficient ionic transport pathways (denoted ion clusters) [7].

The unique nanosize effect (i.e., aerodynamic slip) in nanofibrous materials has led to the successful application of these materials in air filtration [8]. The combination of the large surface areas in nanofibers (NFs) and ionic functional groups has improved the function of the NFs by giving rise to, for example, extremely rapid adsorption and ion exchange kinetics, high adsorption and ion-exchange capacity, and high catalytic activity, leading to a significant expansion of ion-exchange applications [9]. Many studies on the production of Nafion NFs by electrospinning (ES), which is a commonly used method for producing NFs, have been reported [10,11,12,13,14,15,16,17,18]. Particularly, Dong et al. reported a nanosize effect on the ionic conductivity of ion-exchange NFs. The proton conductivity of the prepared electrospun Nafion NFs sharply increased from 0.1 S cm^−1^ in the bulk film to a maximum of 1.5 S cm^−1^ when the fiber diameter was reduced to the nanometer-scale of 400 nm [10]. The conductivity increase is due to the orientation of the ionic domains along the NF axis. This result clearly indicates that control over the internal structure of the NFs during thin fiber formation improves the properties of the ion-exchangers. In addition, Nafion NFs can be used in the form of porous NF mats and/or composites of NF mats and polymer matrices (NF composite membranes) [9]. Such NF networks enable the construction of continuous ion transport pathways in the polymer matrices. Nafion can be dispersed as colloidal particles in a variety of liquids, but the microstructure and rheological properties of the dispersion limit the solution processability, including its spinnability [12,13]. In addition, polymer solutions with high electric conductivity (e.g., polyelectrolyte solution) show low electrospinnability because the high solution conductivity prevents electric field-induced charging of the solution [14]. To enable the stable production of Nafion NFs by ES, water-soluble polymers such as poly(ethylene oxide) (PEO) [15,16], poly(acrylic acid) [17], and poly(vinylpyrrolidone) [18] have been used as spinning aids.

The most serious problem for the practical use of NFs is their low production amount. To solve this issue, scaled-up NF production processes, such as free-surface or multinozzle electrospinning, blowspinning, and centrifugal spinning, have been reported [19]. Table S1 summarizes the advantages and disadvantages of these spinning processes. We think that blowspinning is the most promising for high-throughput production of relatively thin ion-exchange NFs. Solution blow spinning (SBS) was proposed by Mederiou et al. in 2009 [20] and has been developed rapidly in the past decade [21,22]. This process is based on the high-speed stretching of airflow and the Bernoulli principle, by which the change in air pressure is converted into the kinetic energy of the solution. The high-speed airflow generates a shearing force at the gas/solution interface, which deforms the spinning solution from a droplet to a conical shape at the tip of the spinneret. When the shearing force overcomes the surface tension of the solution, a solution jet emerges from the end of the liquid cone and sprays out of the spinneret along the direction of the airflow (see Figure S1, Supplementary Material). The jet flow solvent rapidly evaporates, and the fiber is formed.

In this work, we aim to prepare high-purity perfluorosulfonated ionomer (Nafion) NFs using a high-throughput and scalable NF production process based on high-speed air blowing, SBS, and to characterize the surface and internal structures of the blowspun NFs. The microstructure of the colloidal Nafion dispersion and the corresponding rheological properties are crucial for fiber formation through SBS. In this study, high-molecular weight PEO was used as a spinning aid, and the microstructure and rheological properties of the spinning solutions were evaluated.

## 2. Experimental

### 2.1. Materials and Chemicals

A 20 wt % Nafion^®^ dispersion (DE2020CS type, 34 wt % water, 44 wt % 1-propanol, and 2 wt % other VOCs) was purchased from Fujifilm Wako, Japan. Before use, the Nafion dispersion was vacuum-dried at 40 °C. Polyethylene oxide (PEO) with an average molecular weight of 4,000,000 Da was purchased from Polysciences Inc., United States. Methanol (MeOH, special grade), potassium chloride (KCl, Wako 1st grade), 1 mol L^−1^ hydrochloric acid (HCl, for volumetric analysis), and 0.01 mol L^−1^ potassium hydroxide (KOH, for volumetric analysis) were purchased from Fujifilm Wako, Japan. These reagents were used as received without further purification. Ultrapure water was prepared using a water purification system (Milli-Q Advantage, Merck Millipore, Burlington, MA, USA) and then used as an aqueous solution.

### 2.2. Solution Blow Spinning

The dried Nafion was re-dispersed in MeOH and stirred at 20 °C for 24 h. A small amount of PEO (spinning aid) was added to the Nafion/MeOH dispersion, and the mixture was stirred at 40 °C for 6 h. Thereafter, the solutions were cooled to room temperature and spun. The compositions of the spinning solutions are summarized in Table S2.

**Figure 1:**
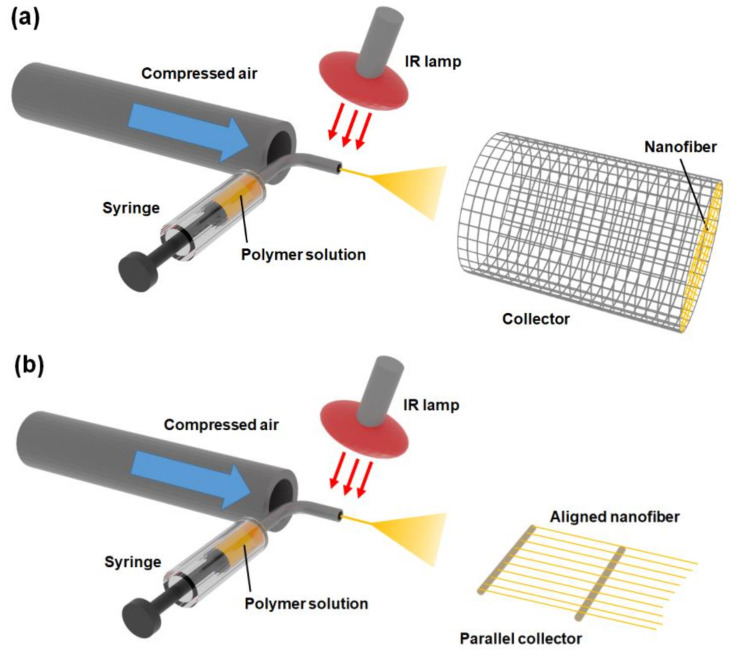
Schematics of (**a**) a basic solution blow spinning (SBS) setup and (**b**) a modified SBS setup for spinning aligned fibers.

A schematic of the SBS setup used here is shown in Figure 1 (a photograph is shown in Figure S1). The spinning solution was contained in a syringe with a stainless-steel nozzle (0.2 mm internal diameter). A constant volume flow rate of 1–5 mL h^−1^ was maintained using a syringe pump (KDS100, KD Scientific Co., Holliston, MA, USA). Compressed dry air (air pressure of approximately 0.1 MPa) was delivered to the nozzle via an oil-free sook roll compressor (SLP-15EFDM5, ANEST IWATA Corporation, Yokohama, Japan). Wire netting was used as the collector. The nozzle-to-collector distance was 300 mm. An IR lamp (100 W, Vivaria, Higashiosaka, Japan) was placed near the nozzle tip to promote solvent evaporation. To produce aligned NFs, two Cu pipes (diameter: 2 mm) were placed parallel to each other and used as the collector (Figure 1b).

For comparison, pure Nafion and Nafion/PEO (99/1) composite films were prepared by casting from 20 wt % Nafion/MeOH and 10 wt % Nafion/0.1 wt % PEO/MeOH dispersions, respectively. The casted samples on the Si wafer and PTFE plate were dried at 20 °C for 6 h and then vacuum-dried at 40 °C for 12 h to obtain the samples for AFM observation and free-standing film (thickness of approximately 40 μm), respectively.

### 2.3. Characterization of Spinning Solutions

The viscosities of the spinning solutions were measured using a rheometer (MCR501, Anton-Paar, Graz, Austria) with a cone plate configuration (CP 50-1, Anton-Paar, Austria) at a shear rate of 10–1000 s^−1^ at room temperature.

Dynamic light scattering (DLS) measurements of the spinning solutions were performed using a Wyatt DynaPro NanoStar (Wyatt Technology, Goleta, CA, USA). All the measurements were performed at room temperature. Before the measurements, the solutions were filtered through a 0.45 μm membrane filter. The hydrodynamic radius (*R*_H_) and diffusion coefficient (*D*) of the polymers in MeOH were calculated based on a CONTIN analysis using DYNAMICS software (Wyatt Technology, USA) [23].

### 2.4. Characterization of NFs

The morphologies of the NFs and films were observed using a scanning electron microscope (SEM, JCM-5700, JEOL, Akishima, Japan) operated at 5 kV. The samples were prepared by sputter coating with Pt. The average fiber diameter and distribution were determined by SEM image analysis using the ImageJ software (NIH, Bethesda, MD, USA). For each NF, at least 100 measurements were carried out. Cross-sectional observations and elemental analyses of the NFs were carried out using a field-emission SEM (FE-SEM, SU9000, Hitachi High-Tech Corp., Tokyo, Japan) equipped with an energy-dispersive X-ray spectrometer (EDS, Genesis, EDAX, Mahwah, NJ, USA) operated at 30 kV. For cross-sectional observations, the NF samples were embedded in epoxy resin.

Potentiometric titration measurements were performed using a potentiometric titrator (888 Titrando, Metrohm, Herisau, Switzerland). The samples (NF or film) were first immersed in a 1 mol L^−1^ HCl solution for 24 h to ensure that the counterions were exchanged with H^+^. After sufficiently washing the samples in ultrapure water, the samples were soaked in a 1 mol L^−1^ KCl solution for 3 h to elute H^+^. Then, the samples were titrated by adding 0.01 mol L^−1^ KOH solution to obtain the titration curves. The amount of fixed charge groups (*N*_x_) in the samples is equal to the titer of KOH. The ion-exchange capacity (IEC) was determined using the equation [24]

(1) $$\mathrm{IEC}=\frac{N_{x}}{w_{\mathrm{dry}}}$$

where *w*_dry_ is the weight of the sample in the dry state. The samples were vacuum-dried at 40 °C for 12 h, and the dry weight was determined.

The water swelling behavior of the samples was characterized under humid conditions (20 °C, 70% RH). The sample weight in the equilibrium swollen state (*w*_wet_) was measured. Subsequently, the samples were vacuum-dried at 40 °C for 12 h and *w*_dry_ was determined. The water content of the samples *w*_w_ is defined as [24]

(2) $$w_{w}=\frac{w_{\mathrm{wet}}-w_{\mathrm{dry}}}{w_{\mathrm{dry}}}\;\times \;100.$$

Height and phase images of the samples were acquired using an atomic force microscope (AFM, MFP-3D, Asylum Research, Goleta, CA, USA) in tapping mode in the attractive regime. All the observations were carried out at 25 °C and 40% RH under ambient conditions. For the observation, a single NF was fixed on the Si substrate, and then both sides were fixed using adhesive (see Figure S2).

Small-angle X-ray scattering (SAXS) measurements were performed at the B40L beamline at SPring-8 (Hyogo, Japan). The aligned NF samples were irradiated with X-rays of wavelength *λ* = 0.1 nm. The scattering patterns were recorded on a PILATUS3 S 2M detector (Dectris, Baden-Daettwil, Switzerland) located at a distance of 2264 mm from the sample.

Wide-angle X-ray diffraction (WAXD) measurements were carried out using a diffractometer (UltraX 18HB, Rigaku, Akishima, Japan) with Cu-Kα radiation. The degree of crystallinity (*X*_c_) was calculated as the ratio of the crystalline peak area to the total area under the scattering curve using the equation [25]

(3) $$X_{c}\left(\%\right)=\frac{I_{c}}{I_{c}+I_{a}}\times 100$$

where *I*_c_ and *I*_a_ are the integrated intensities from crystal diffraction and amorphous scattering, respectively.

The apparent crystallite size (*τ*) was analyzed using the Scherrer equation [25]

(4) $$\tau \left(Å\right)=\frac{k\lambda }{\beta \cos\theta }$$

where λ is the X-ray wavelength (1.5416 Å), *k* is the dimensionless shape factor (*k* = 0.9), *θ* is the Bragg angle, and *β* is the full width at half the maximum intensity (FWHM) of the diffraction peak.

## 3. Results and Discussion

### 3.1. Characterization of Spinning Solutions

Before preparing the NFs, we measured the rheological properties of the spinning solutions used in this study. The obtained results for the shear rate dependence of the viscosity are shown in Figure S3. The specific viscosity (*η*_sp_) was calculated from the experimental results shown in Figure S3 as

(5) $$\eta_{\mathrm{sp}}=\frac{\eta_{0}-\eta_{s}}{\eta_{s}}$$

where *η*_0_ is the zero-shear viscosity (we determined the values by extrapolation of the viscosity-shear rate plots in Figure S3), and *η*_s_ is the viscosity of the solvent (MeOH).

**Figure 2:**
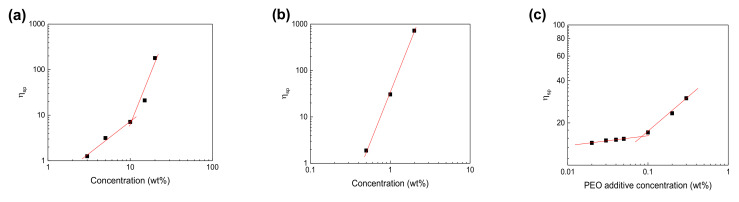
Dependence of specific viscosity on concentration of (**a**) Nafion/MeOH dispersions, (**b**) PEO/MeOH dispersions, and (**c**) 10 wt % Nafion-PEO/MeOH solutions.

Figure 2 shows the dependence of the specific viscosity (*η*_sp_) on the concentration (*C*) of the spinning solutions. For the Nafion/MeOH dispersions (Figure 2a), the critical overlap concentration (C*) is approximately 10 wt %. The scaling relationships take the forms of *η*_sp_ ≈ *C*^1.49^, and *η*_sp_ ≈ *C*^4.53^ in the low and high *C* regimes, respectively. The former and latter respectively correspond to the theoretically predicted semi-dilute unentangled (*η*_sp_ ≈ *C*^1.25^) and semi-dilute entangled (*η*_sp_ ≈ *C*^4.8^) regimes for neutral linear polymers in a good solvent [13,26]. Although we could not blowspin all the pure Nafion/MeOH dispersions (see Table S1), including the solution with a concentration larger than the C* of 10 wt %, these results are consistent with previous ES work [16]. For the PEO/MeOH solutions (Figure 2b), the relationship takes the form of *η*_sp_ ≈ *C*^0.50^, which corresponds to the dilute regime because the exponent of *C* is lower than 1.25. The blowspinning of PEO/MeOH solutions was unstable, but fibrous structures were formed for the solutions with *C* > 1.0 wt % (see Table S1). In previous ES work, partial NF formation from high-molecular weight polymer solutions with *C* < C* was reported. Similarly, the contribution of high-molecular weight PEO in SBS is also more substantial [27]. The C* of Nafion (10 wt %)–PEO/MeOH solutions with various PEO additive concentrations (Figure 2c) is 0.09 wt %. The *C* of Nafion in the composite solutions is in the entangled semi-dilute regime (see Figure 2a). The rapid increase in *η*_sp_ beyond 0.09 wt % PEO concentration reflects the microstructural change in the solution.

**Figure 3:**
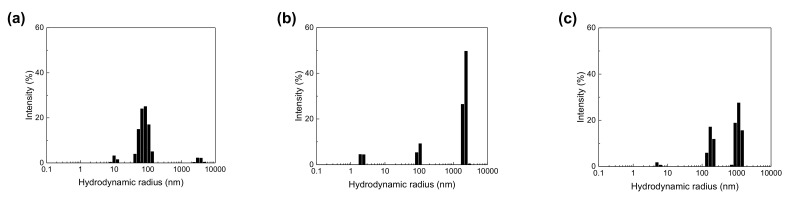
Hydrodynamic radius (*R*_H_) distribution determined by DLS measurements of (**a**) 0.1 wt % PEO/MeOH solution, (**b**) 10 wt % Nafion/MeOH dispersion, and (**c**) 10 wt % Nafion–0.1 wt % PEO/MeOH solution.

DLS measurements were conducted to evaluate the microstructure of the spinning solutions. Figure 3 shows the hydrodynamic radius (*R*_H_) determined by DLS measurements. For the 0.1 wt % PEO/MeOH solution, the main peak indicates a structure with a *R*_H_ between 50 and 100 nm (Figure 3a), which is comparable to that of a random coil of PEO chains with a *M*_w_ of 4,000,000 in *θ* solvent [28]. In the 10 wt % Nafion/MeOH dispersion, there are three peaks corresponding to the *R*_H_ values of 1–5 nm, approximately 100 nm, and >1 μm (Figure 3b). The peak at the smallest value corresponds to the *R*_H_ of singular molecular chains, while the latter two peaks correspond to primary (rod-like particles) and secondary aggregates, respectively [27,29]. The microstructure is different from that of dilute Nafion/MeOH dispersions [29]. The scattering intensity reveals that most of the Nafion chains formed secondary aggregates. In contrast, in the 10% Nafion-0.1 wt % PEO/MeOH solution, which has the same composition as the optimized spinning solution composition described later, the addition of a small amount of high-molecular weight PEO substantially decreased both the size and number of secondary aggregates, and the number of primary aggregates. This change in microstructure induced by the addition of PEO is probably due to the changes in the ionic strength of the spinning solution [17] and/or interaction between the sulfonic acid groups of Nafion and PEO [30]; however, the disaggregation mechanism requires further investigation.

### 3.2. Preparation of NFs

**Figure 4:**
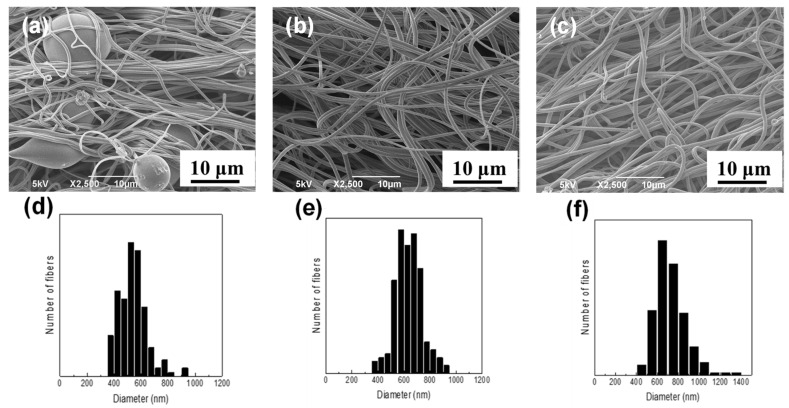
Typical surface SEM images and fiber diameter distributions of the randomly-deposited NFs prepared by SBS from 10 wt % Nafion/MeOH solutions containing PEO at the flow rate of 1.0 mL h^−1^. The compositions of the Nafion/PEO composite NFs are (**a**) 99.5/0.5, (**b**) 99/1, and (**c**) 98.5/1.5. (**d**–**f**) Corresponding fiber diameter distributions of parts (**a**–**c**), respectively, from the SEM image analysis. For each NF, at least 100 measurements were carried out.

Fibrous materials could not be produced by SBS from the 5–20% Nafion/MeOH dispersions without a spinning aid (see Table S1). One possible reason is because the microscale higher-order agglomerates in the concentrated Nafion dispersions prevented stable fiber formation (see Figure 3b). Thus, by adding a small amount of PEO to the dispersions, we successfully produced Nafion NFs by SBS. Figure 4 shows typical SEM images of the surface and the diameter distributions of the blowspun NFs produced from 10 wt % Nafion/MeOH solutions containing various contents of PEO at a flow rate of 1.0 mL h^−1^. For the PEO concentration of 0.05 wt %, beaded fibers were obtained, while bead-free and smooth fibers were produced at PEO concentrations of 0.5% or higher. The average fiber diameters obtained from the SEM image analysis are 540 ± 104 nm, 640 ± 103 nm, and 720 ± 143 nm for 10 wt % Nafion/MeOH solutions containing the PEO concentrations of 0.05 wt %, 0.10 wt %, and 0.15 wt %, respectively, indicating that the diameter increases with the amount of added PEO. These findings clearly indicate that the addition of PEO disaggregates higher-order Nafion agglomerates, consequently improving the spinnability (see Figure 3c) and finally leading to the production of stable, bead-free, and smooth high-purity NFs. In addition, by controlling the flow rate of the spinning solution, bead-free and smooth NFs could also be produced by SBS from the 10% Nafion-0.1 wt % PEO/MeOH solution at higher flow rates (3.0 mL h^−1^ and 5.0 mL h^−1^, see Figure S3), indicating the potential for scaling up.

Here, we used the thinnest randomly deposited NFs with a bead-free and smooth structure, which were blowspun from 10 wt % Nafion–0.1 wt % PEO/MeOH solution, for the next characterization. In order to investigate the orientation of internal structure of the NFs, the highly aligned NFs were prepared (Figure S4 and Table S3) and used for X-ray analysis.

### 3.3. Properties and Surface/Internal Structures of NFs

**Figure 5:**
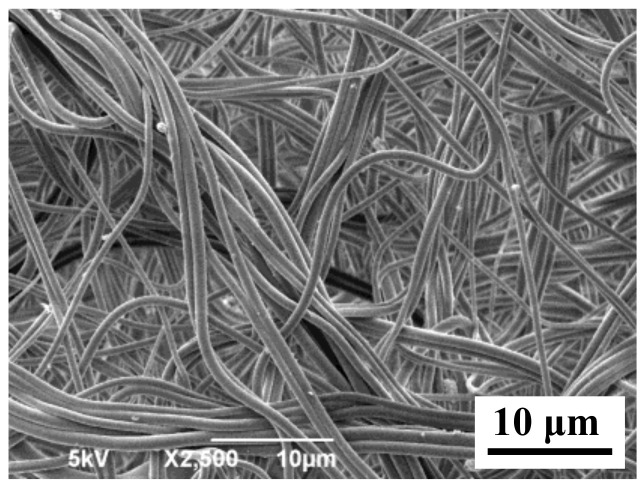
SEM image of Nafion/PEO (99:1) composite NFs after immersion in water for 1 day.

The physicochemical properties of the prepared NFs are listed in Table 1. The ion-exchange capacity (IEC) and water content (*w*_w_) at 70% RH of the prepared NF compare favorably with those of the as-cast Nafion film (IEC of approximately 1.0 mmol g^−1^ and *w*_w_ of approximately 8%). The dimensions of the NFs were maintained even after they were immersed in water for 1 d (see Figure 5). The excellent water resistance of the NFs is due to the high content of Nafion. The porosity (*ε*) of the randomly deposited NF mat was calculated using the apparent density of the mat ($\rho_{i}$) and the densities of the as-cast Nafion/PEO (99:1) film ($\rho_{0}$) using the following equation [25]:

(6) $$\varepsilon =\left(1-\frac{\rho_{i}}{\rho_{0}}\right)\times 100.$$

The prepared Nafion NF mat exhibited high porosity of 87.3%.

**Figure 6:**
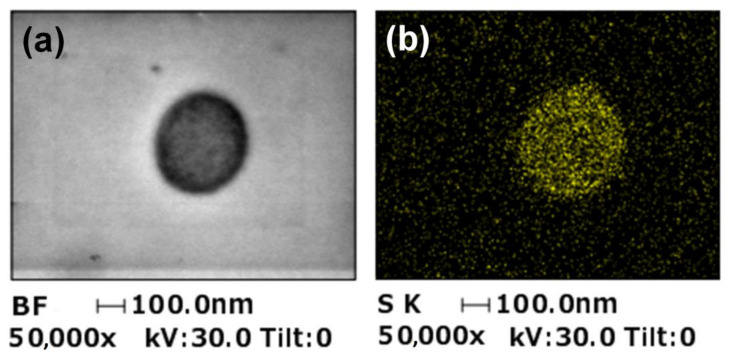
Cross-sectional STEM-EDS images of a single Nafion/PEO (99:1) composite NF. (**a**) Cross-sectional bright-field (BF) STEM image and (**b**) EDS mapping of sulfur atoms in the NF.

To investigate the distribution of the sulfonic acid groups in the NFs, cross-sectional STEM/EDS analysis was conducted. EDS mapping of the sulfur atoms revealed that the sulfonic acid groups were homogeneously distributed within the NFs (Figure 6b).

**Figure 7:**
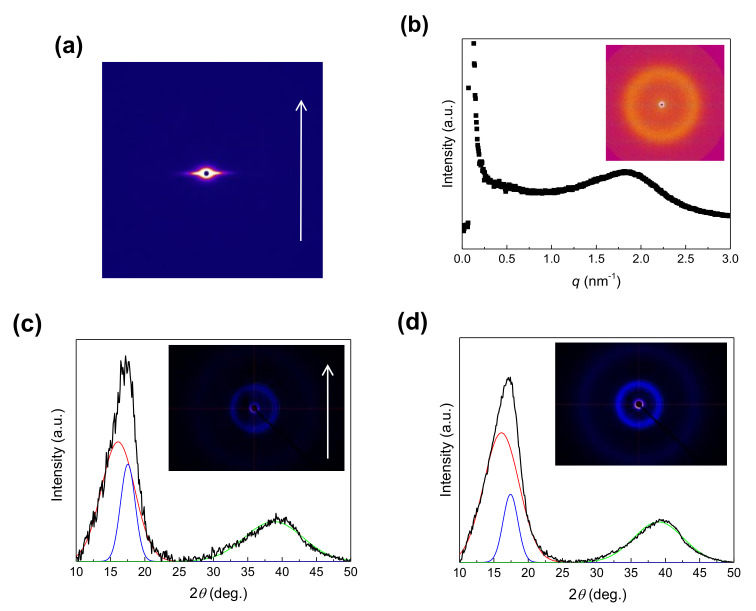
SAXS and WAXD results of Nafion/PEO (99:1) composite samples. (**a**) Two-dimensional (2D) SAXS pattern of the aligned NFs and (**b**) SAXS profile of the film. (Inset) 2D SAXS pattern. De-convoluted WAXD profiles of the (**c**) aligned NFs and (**d**) film. (Insets) 2D WAXD patterns. The white arrows in (**a**,**c**) show the fiber axis direction.

SAXS and WAXD measurements were performed to characterize the internal microstructures of the aligned NFs. The obtained data are shown in Figure 7. The NFs do not show a clear SAXS scattering pattern (Figure 7a), but the thin films show a ring-shaped pattern with a peak at the scattering vector *q* = 1.84 nm^−1^ (the corresponding spacing *d* = 2π/*q* = 3.41 nm) (Figure 7b). The peak in the film (denoted as the ionomer peak) corresponds to the center-to-center distance between the hydrophilic domains in Nafion [5,31,32]. Microphase separation between the hydrophilic and hydrophobic domains in the NFs was suppressed, presumably because of the high extensional strain rate and rapid solidification during SBS.

The 2D WAXD patterns for both the NFs and the films show two broad diffraction peaks. The peaks in the WAXD profiles were deconvoluted using a Gaussian function to determine the degree of crystallinity (*X*_c_) and apparent crystallite size (*τ*). The NFs exhibit three peaks at the 2*θ* values of 16.1°, 17.5°, and 38.8° (Figure 7c), corresponding to the intermolecular correlations in the amorphous phase [33], while the (100) planes were derived from 15/7 helical formation of CF_2_−CF_2_ chains [6], and the amorphous phase, respectively. Similar peaks are observed in the profile of the films. Table 2 summarizes the *X_c_* and *τ* values calculated from the WAXD data for both the NFs and the films. The *X_c_* values for the NFs and films are 18.5% and 13.4%, respectively, which are comparable to the reported values for Nafion (10–20%) [6]. Note that the NFs are more crystalline than the films, and their crystallite size is larger than that of the films. The enhanced crystallization of the CF_2_−CF_2_ chains in the Nafion NFs may be ascribed to the higher extensional strain rate experienced by the individual fibers during SBS. The enhanced crystallization may also be related to the suppression of microphase separation.

**Figure 8:**
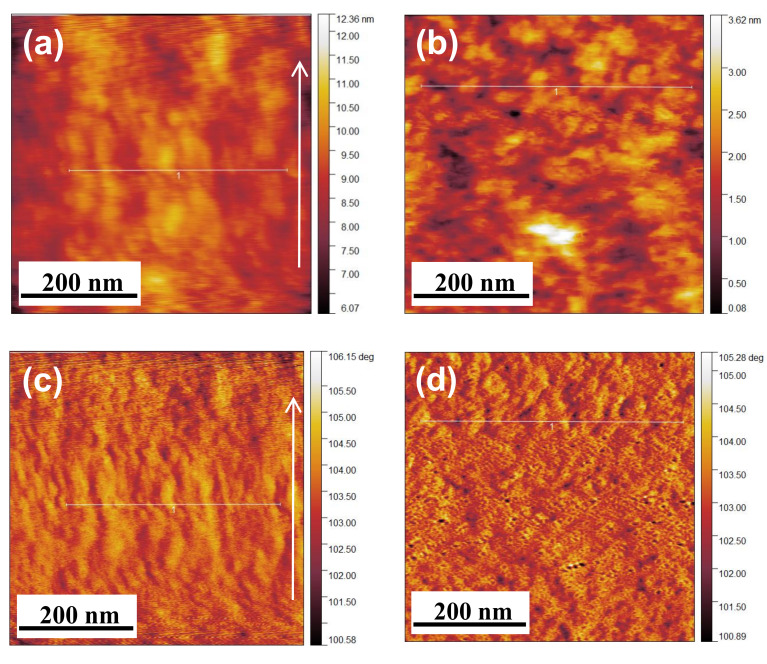
AFM data of Nafion/PEO(99:1) composites. Topology images of the (**a**) NF and (**b**) film. Phase images of the (**c**) NF and (**d**) film. The white arrows show the fiber axis direction. All observations were carried out at 25 °C and 40% RH. The line profiles of all the images are shown in Figure S6.

To clarify the phase separation between the hydrophilic and hydrophobic domains on the blowspun NF surface, topological and phase images of the NF surface were next recorded with an AFM in tapping mode under ambient conditions (see Figure S2). The dark and bright regions in the phase images in Figure 8 are derived from the hydrophilic (ionic) and hydrophobic domains of Nafion, respectively. (In the attractive mode, the darker areas correspond to larger power dissipations relative to the brighter areas [34].) For the film, many dark nanoscale circular areas were observed. These areas were due to microphase separation. For the NF, in contrast, the surface structures were not well ordered, and incompletely segregated dark and bright areas were observed. Note that both areas on the NF surface were oriented along the fiber axis. This less-ordered structure does not contradict the internal structure obtained by the SAXS analysis, and the oriented regions could also be due to the high extensional strain rate and/or rapid solidification during blow spinning. (The micelle orientation at the free-surface was also reported for the spin-coated Nafion films with centrifugal force and rapid solidification [35]). Dong et al. reported the formation and higher orientation of hydrophilic domains (ionic aggregates) in thinner electrospun Nafion NFs [10]. The difference in the structures between blow-spun and electrospun NFs could be due to the driving force of spinning. Note that the solidification of the polymer liquid jet during SBS with the use of pressurized gas is faster than that during ES with the use of an electric field.

## 4. Conclusions

In this work, we prepared high-purity Nafion (99%) NFs by SBS and investigated their surface and internal structures. The structures of the blowspun Nafion NFs were different from those of the electrospun NFs: the microphase separation between the hydrophilic and hydrophobic domains was suppressed, but the crystallization of the CF_2_−CF_2_ chains was enhanced. These differences may be caused by the high extensional strain rate and rapid solidification during SBS. The speed of SBS is faster than that of ES, and the electrical parameters of the polymer solution are much less limited [22]. To the best of our knowledge, this is the first report on preparation of high-purity Nafion NFs by SBS. In addition, we have revealed that the surface and internal structures of the blowspun NFs are different from those of casted films and electrospun NFs. These findings clearly indicate a novel protocol for the microstructure control of ionomers: different driving forces of spinning lead to different surface and internal structures of the NFs. In addition, SBS is more suitable for high-throughput spinning of polyelectrolyte solutions and polymer solutions containing large amounts of electrolytes. We believe that high-purity polyelectrolyte (ion-exchange) NFs produced by high-throughput and scalable processes can be industrialized for applications such as fuel cells, catalysts, water electrolysis, electrodialysis, reverse electrodialysis, and capacitive deionization in the forms of porous NF mats and/or composites of NF mats and polymer matrices (NF composite membranes) [9].

## Figures and Tables

**Table 1:** Physicochemical properties of the prepared Nafion/PEO composite NFs.

Samples	IEC [mmol g^−1^]	*w*_w_* [%]
Nafion/PEO (99:1) NF	1.0	7.5
As-cast Nafion film	1.1	7.8

* Equilibrated at 20 °C and 70%RH for 5 h.

**Table 2:** Degree of crystallinity and apparent crystallite size of the aligned NFs and Nafion/PEO (99:1) composite samples.

Samples	Crystalline Peak (°)	Amorphous Peak		*X*_c_ *^a^* (%)	*τ ^b^* (nm)
		#1 (°)	#2 (°)		
Nafion/PEO (99:1) NF	17.5	16.1	38.8	18.5	5.46
Nafion/PEO (99:1) film	17.4	16.1	39.0	13.4	5.37

*^a^* Degree of crystallinity. *^b^* Apparent crystallite size.

## Supplementary Materials

The following are available online at https://www.mdpi.com/article/10.3390/membranes11060389/s1, Figure S1: Photograph of the solution blow spinning setup. (Inset) Jet of the polymer solution formed near the tip of the nozzle, Figure S2: Optical micrographs of (a) a blowspun single Nafion/PEO (99:1) composite nanofiber, (b) a single nanofiber fixed on a Si substrate, (c) AFM cantilever and the fixed single nanofiber, Figure S3: Dependence of viscosity on shear rate for (a) Nafion/MeOH dispersions, (b) PEO/MeOH dispersions, and (c) 10 wt % Nafion-PEO/MeOH solutions at various concentrations, Figure S4: Typical SEM images of the blowspun NFs prepared from 10 wt % Nafion/MeOH solutions containing 0.1 wt % PEO (Nafion/PEO = 99/1). The flow rates of spinning solutions were (a) 3 mL h^−1^ and (b) 5 mL h^−1^, Figure S5: (a) Typical SEM image and (b) fiber angle histogram (*N* = 200) of the aligned Nafion/PEO (99:1) composite nanofibers, Figure S6: Line profiles of the white lines in the AFM images shown in Figure 8. Topology profiles of (a) the NF and (b) the film. Phase profiles of (c) the NF and (d) the film, Table S1: Comparison of spinning processes, Table S2: Components of spinning solutions for SBS, Table S3: Fiber angle of the aligned Nafion/PEO (99:1) composite nanofibers (*N* = 200).
