# Supplementary material for: Preparation of Perfluorosulfonated Ionomer Nanofibers by Solution Blow Spinning
Source: Membranes (Basel). 2021 May 25;11(6):389. doi: 10.3390/membranes11060389 (PMC8228795; doi:10.3390/membranes11060389)
Supplement: Supplementary file 1 [file membranes-11-00389-s001.zip › membranes-1217269-supplementary.pdf]

membranes

Supplementary Material: Preparation of Perfluorosulfonated Ionomer Nanofibers by Solution Blow Spinning

Masahiro Shinkawa, Kazunori Motai, Keita Eguchi, Wataru Takarada, Minoru Ashizawa, Hiroyasu Masunaga, Noboru Ohta, Yuhei Hayamizu and Hidetoshi Matsumoto

Table 1. Comparison of spinning processes [1,2].

	Solution blow spinning	Electrospinning	Centrifugal spinning
Single-nozzle productivity	0.5–1 g/h	1–100 mg/h	1–50 g/h
Productivity in other mode	400 g/h (multi-nozzles)	278 g/h (nozzle-less)	500 g/h (multi-nozzles)
Dimensions	100–1000 nm	10–50 nm	> 1 μm
Advantages	No voltage<br>Good industrialization prospects		No voltage<br>Controllable deposition
Disadvantages	Random deposition (large scale)	High voltage<br>Preparation of polyelectrolyte is difficult	Larger diameter

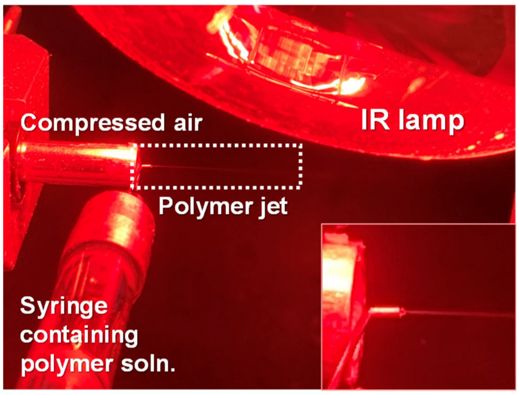

Figure S1. Photograph of the solution blow spinning setup. (Inset) Jet of the polymer solution formed near the tip of the nozzle.

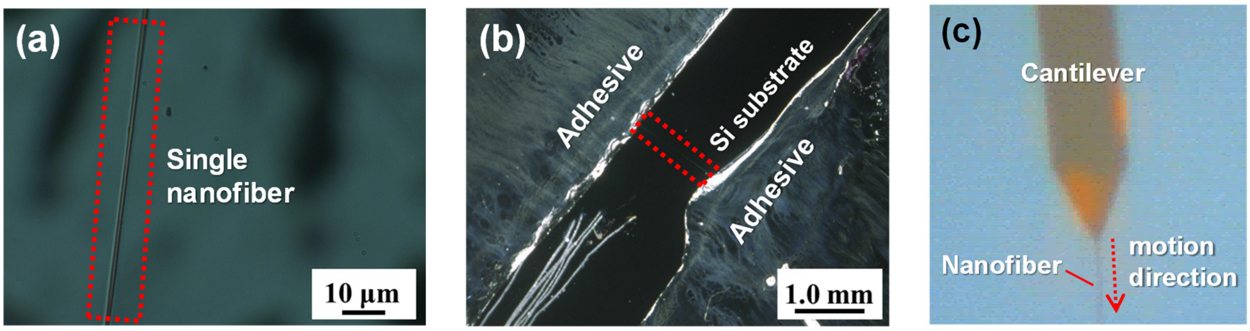

Figure S2. Optical micrographs of (**a**) a blowspun single Nafion/PEO (99:1) composite nanofiber, (**b**) a single nanofiber fixed on a Si substrate, (**c**) AFM cantilever and the fixed single nanofiber.

For the Nafion/MeOH dispersions (3–20 wt%, Figure S3a), the 20 wt% Nafion dispersion showed a gel-like behavior with a rapid increase in viscosity in the low shear rate region [3] while all the other solutions showed almost constant viscosity over a wide range of shear rates. For the PEO/MeOH solutions (0.3–2.0 wt%, Figure S3b, we could not measure the viscosity of the solutions with $C \leq 0.2$ wt%), the viscosity of the 2.0 wt% solution increased rapidly in the low shear rate region, indicating a gel-like behavior while the 1.0 wt% solution showed a shear thinning behavior in which the viscosity decreased with increasing shear rate. For the Nafion-PEO/MeOH solutions (Nafion concentration was fixed at 10 wt%, which is same as the optimized composition of the spinning solutions, and the PEO concentration was varied from 0.02–0.30 wt%, Figure S3c), all solutions showed a shear thinning behavior in which the viscosity decreased with increasing shear rate.

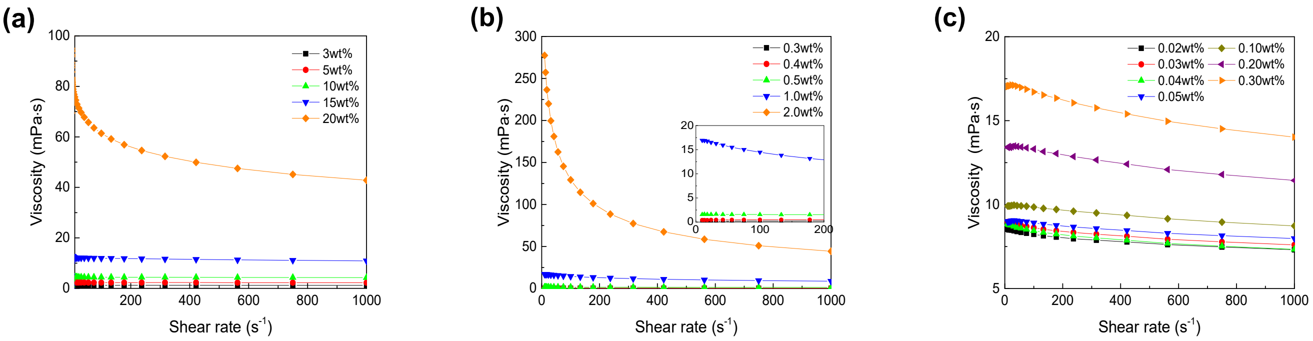

Figure S3. Dependence of viscosity on shear rate for (**a**) Nafion/MeOH dispersions, (**b**) PEO/MeOH dispersions, and (**c**) 10 wt% Nafion-PEO/MeOH solutions at various concentrations.

Table 2. Component of the spinning solutions for SBS.

**Sample No.**	Component			Morphology of product
	Nafion (wt%)	PEO (wt%)	MeOH (wt%)	
1	5	0	95	N.A.
2	10	0	90	N.A.
3	15	0	85	N.A.
4	20	0	80	N.A.
5	10	0.05	89.95	Beaded-fiber
6	10	0.10	89.90	Fiber
7	10	0.15	89.85	Fiber
8	15	0.05	84.95	Fiber
9	15	0.10	84.90	Fiber
10	20	0.05	79.95	Fiber
11	20	0.10	79.90	Fiber
12	0	0.5	99.5	N.A.
13	0	1.0	99.0	Fiber
14	0	1.5	98.5	Fiber

N.A.: Stable polymer jet could not be formed.

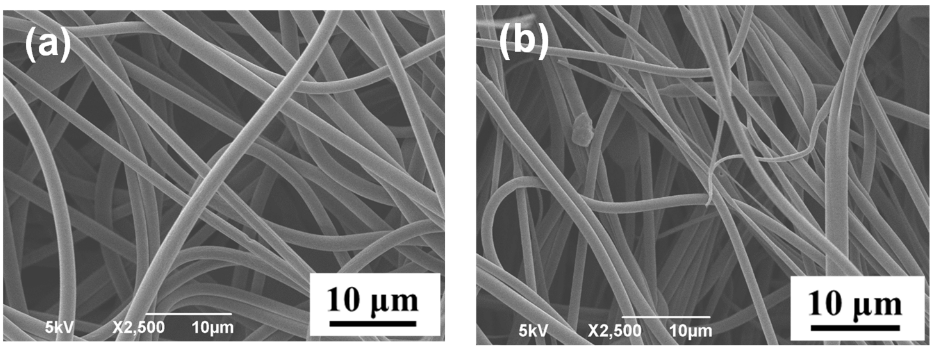

Figure S4. Typical SEM images of the blowspun NFs prepared from 10 wt% Nafion/MeOH solutions containing 0.1 wt% PEO (Nafion/PEO=99/1). The flow rates of spinning solutions were (**a**) 3 mL h^{-1} and (**b**) 5 mL h^{-1}.

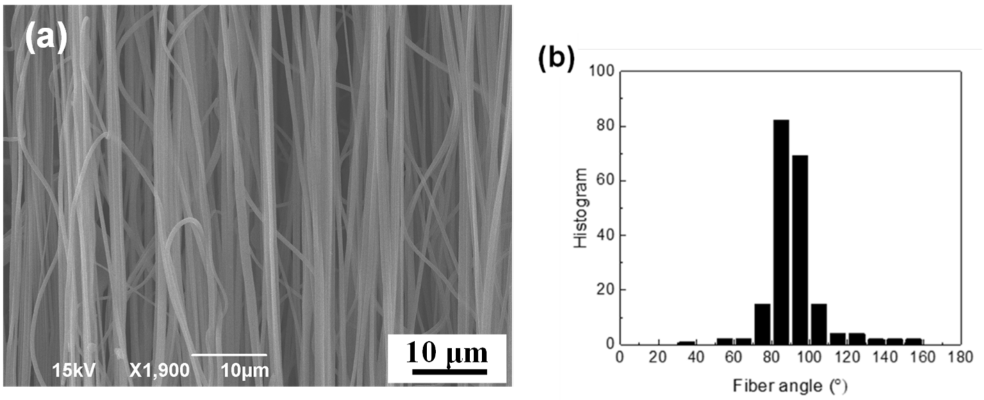

Figure S5. (**a**) Typical SEM image and (**b**) fiber angle histogram (N = 200) of the aligned Nafion/PEO (99:1) composite nanofibers.

Table S3 Fiber angle of the aligned Nafion/PEO (99:1) composite nanofibers (N = 200).

**Fiber angle range (°)**	**Number of fibers**	**Percentage (%)**
±5°	120	60
±10°	151	76
±20°	181	91
±30°	187	94

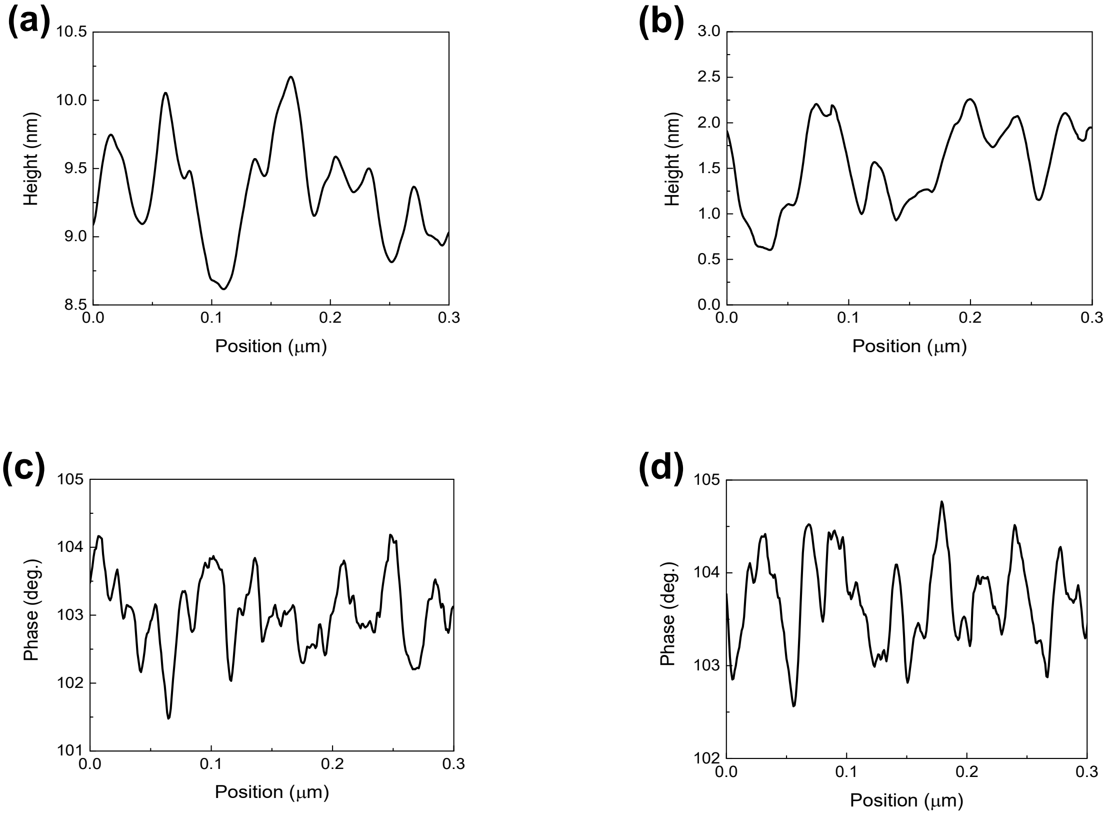

Figure S6. Line profiles of the white lines in the AFM images shown in Figure 8. Topology profiles of (a) the NF and (b) the film. Phase profiles of (c) the NF and (d) the film.

References

1. Huang, Y.; Song, J.; Yang, C.; Long, Y.; Wu, H. Scalable manufacturing and applications of nanofibers. *Mater. Today* **2019**, *28*, 98–113, doi:10.1016/j.mattod.2019.04.018.
2. Gao, Y.; Zhang, J.; Su, Y.; Wang, H.; Wang, X.-X.; Huang, L.-P.; Yu, M.; Ramakrishna, S.; Long, Y.-Z. Recent progress and challenges in solution blow spinning. *Mater. Horiz.* **2021**, *8*, 426–446, doi:10.1039/D0MH01096K
3. Dong, B.; Gwee, L.; Cruz, D.S.; Winey, K.I.; Elabd, Y.A. Super proton conductive high-purity Nafion. *Nano Lett.* **2010**, *10*, 3785–3790, doi:10.1021/nl102581w.
